# Enigmatic Pilus-Like Endospore Appendages of *Bacillus cereus* Group Species

**DOI:** 10.3390/ijms222212367

**Published:** 2021-11-16

**Authors:** Ephrem Debebe Zegeye, Brajabandhu Pradhan, Ann-Katrin Llarena, Marina Aspholm

**Affiliations:** 1 Department of Paraclinical Sciences, Faculty of Veterinary Medicine, Norwegian University of Life Sciences (NMBU), P.O. Box 5003, 1432 Ås, Norway; ephremdebebe@gmail.com (E.D.Z.); ann-katrin.llarena@nmbu.no (A.-K.L.); 2 Structural and Molecular Microbiology, VIB-VUB Center for Structural Biology, VIB, 1050 Brussels, Belgium; Brajabandhu.Pradhan@vub.be; 3 Department of Bioengineering Sciences, Structural Biology Brussels, Vrije Universiteit Brussel, 1050 Brussels, Belgium

**Keywords:** endospore, spore, pili, appendage, *Bacillus cereus*, Ena

## Abstract

The endospores (spores) of many *Bacillus cereus* sensu lato species are decorated with multiple hair/pilus-like appendages. Although they have been observed for more than 50 years, all efforts to characterize these fibers in detail have failed until now, largely due to their extraordinary resilience to proteolytic digestion and chemical solubilization. A recent structural analysis of *B. cereus* endospore appendages (Enas) using cryo-electron microscopy has revealed the structure of two distinct fiber morphologies: the longer and more abundant “Staggered-type” (S-Ena) and the shorter “Ladder-like” type (L-Ena), which further enabled the identification of the genes encoding the S-Ena. Ena homologs are widely and uniquely distributed among *B. cereus* sensu lato species, suggesting that appendages play important functional roles in these species. The discovery of *ena* genes is expected to facilitate functional studies involving Ena-depleted mutant spores to explore the role of Enas in the interaction between spores and their environment. Given the importance of *B. cereus* spores for the food industry and in medicine, there is a need for a better understanding of their biological functions and physicochemical properties. In this review, we discuss the current understanding of the Ena structure and the potential roles these remarkable fibers may play in the adhesion of spores to biotic and abiotic surfaces, aggregation, and biofilm formation.

## 1. Introduction

Many bacterial species belonging to the phylum *Firmicutes*, including *Bacillus* spp. and *Clostridium* spp., can differentiate into the extremely resilient endospore (spore) form. Environmental stressors, such as nutrient deprivation, dehydration, and/or suboptimal temperature, trigger the onset of sporulation in *Bacillus* spp. Spores are extremely hardy and metabolically inert structures that can withstand harsh environmental insults, including freezing, desiccation, UV irradiation, extreme heat, and exposure to toxic chemicals. They are also known to survive phagocytosis and escape from macrophages [1]. Spores constitute an extreme form of dormancy that allows life to remain latent for long periods of time until favorable environmental stimuli trigger their germination and outgrowth [2].

Spore-forming bacteria pose a formidable challenge to food safety, quality, and sustainability due to their ubiquitous presence in many types of food and, more importantly, their inherent resistance to destruction by cleaning agents, pasteurization, and cooking temperatures. Endospore-forming bacteria of special public health and food safety interest include *B. cereus* sensu lato (s.l.) (*B. cereus*/*B. anthracis*/*B. thuringiensis* family),* B. sporothermodurans*, *Clostridium botulinum*, *C. perfringens*, and *Clostridiodes difficile*.

As spores adhere tightly to inorganic surfaces, they pose a challenge to the food industry by sticking to machines and pipes [3,4,5]. Attached spores can survive cleaning-in-place (CIP) procedures, leading to the accumulation of microorganisms in food production equipment and on surfaces over time [5,6]. Interestingly, spores that adhere to surfaces such as stainless steel and rubber have increased heat resistance compared to their planktonic counterparts [7,8]. To make matters worse, under favorable conditions, adhered spores may germinate and outgrow into vegetative bacteria that can begin to form a biofilm (alone or mixed with other bacterial species) [4,9,10]. Biofilms provide vegetative bacteria and spores with an extra protective exopolysaccharide matrix that can further undermine the efficiency of cleaning procedures and cleaning reagents [11]. Established biofilms are difficult to eradicate and can lead to persistent contamination of food products [11]. Similarly, *C. difficile* adheres to various inorganic surfaces in hospitals, resulting in a persistent risk of nosocomial infections [12,13,14]. Collectively, the characteristics that allow endospores to remain persistently in different environments are a major public health and sustainability problem [9,10,15,16,17]. Therefore, a better understanding of their structure, physiology and adhesion properties may facilitate the development of more effective strategies for successful inactivation and/or removal of endospores.

## 2. *Bacillus* Endospore Structure

**Figure 1:**
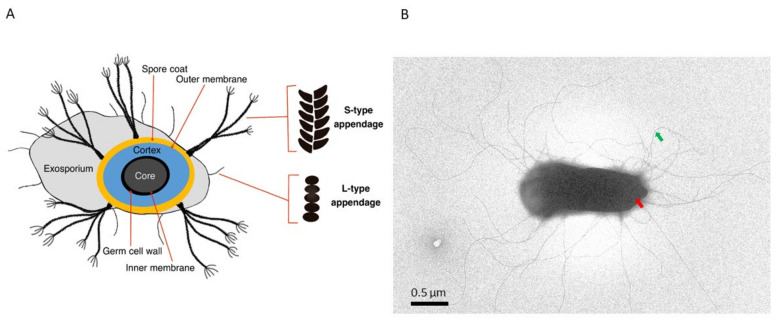
*B. cereus* endospore decorated with multiple hair-like appendages. (**A**) Illustration showing a cross-section of a *B. cereus* spore revealing multiple concentric layers, the exosporium and endospore appendages (S-Ena and L-Ena). The sizes of the spore layers and appendages are not drawn to scale. (**B**) Negative stain transmission electron microscopy (TEM) image of *B. cereus* NVH0075-95 spore showing the spore body (red arrow) and hair (pilus)-like appendages (S-Ena) (green arrow).

An insight into the spore architecture and biochemical composition uncovers not only the armors they are equipped with to survive under harsh conditions, but also the receptors that enable them to detect environmental stimuli, and under favorable signals, germinate within minutes [2,18]. Typically, spores have a core surrounded by an inner membrane, a germ cell wall (also known as a core wall), a cortex, an outer spore membrane, and a coat layer [19] (Figure 1A). Thin-section transmission electron microscopy (TEM) examination of endospores reveals concentric multilayers that encase and safeguard the core. The core predominantly contains the bacterial genome, proteins, ribosomes, and RNA. It is partially dehydrated and contains large amounts of Ca^2+^-dipicolinic acid and small acid-soluble proteins that protect the genome against damage [20], for example, caused by ionizing [21] and non-ionizing radiation [22]. The proteins in the core are stabilized by rotational immobilization, a potential mechanism that minimizes irreversible aggregation of the proteins after exposure to extreme heat [23]. Receptors located in the inner membrane interact with germinant molecules (e.g., amino acids, sugars, inorganic salts, and purine nucleosides), triggering a cascade of events that eventually lead to germination and outgrowth of the vegetative cell [2]. The cortex layer is essential for maintaining the dehydrated state and dormancy of the spores. The multilayered proteinaceous coat is composed of more than 70 different proteins, and it represents the outermost layer of spores of many *Bacillus* species, e.g., *B. subtilis* group spp. [24]. The number of coat layers, their thickness, and biochemical composition vary between species [18]. In some spore-forming species, such as *B. cereus*, *B. anthracis*, *B. thuringiensis,* and *B. mycoides*, the coat is enclosed by a loosely fitting balloon-like structure known as the exosporium. In *B. cereus*, the adhesion of the spores to surfaces such as host cells [25] and stainless steel has been attributed to the exosporium layer [26]. Details on the structure of the endospore coat, the exosporium, and their assembly and functions are reviewed elsewhere [18,27]. Interestingly, the exosporium of most *B. cereus* spp. endospores is decorated with multiple hair or pilus-like appendages [27,28] whose biological functions have not yet been elucidated (Figure 1B). A recent structural analysis of endospore appendages of *B. cereus* revealed that they represent a novel superfamily of pili that exhibit extreme physicochemical sturdiness, probably reflecting an adaptation to the harsh conditions that spores likely encounter in different environments [29]. In this review, we summarize the current state of knowledge on these mysterious pilus-like fibers in light of the recent advances in the genetic and structural characterization of Enas [29], and further outline the potential roles they play in the biology of *B. cereus* spp.

## 3. Endospore Appendages

**Figure 2:**
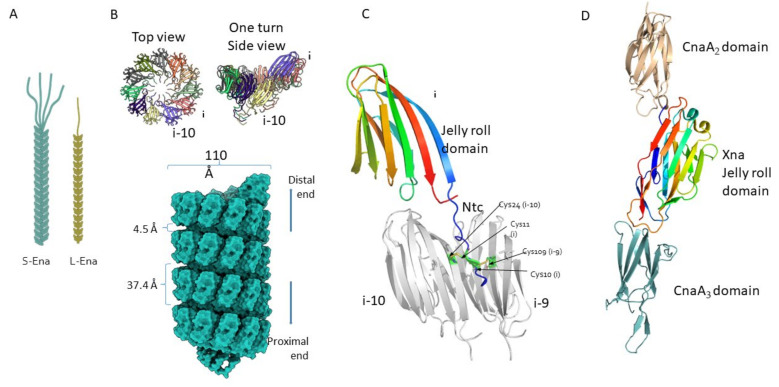
Architecture of the Ena fibers. (**A**) Illustration of S-Ena and L-Ena fibers with staggered and ladder-like arrangements of Ena subunits, respectively. In their termini distal to the endospore, the S-Ena fiber has ~4–5 ruffles (thin filamentous extensions of ~4 nm diameter), while the L-Ena has a single ruffle. (**B**) Surface representation of the atomic model of four helical turns of the S-Ena. Side and top view of one S-Ena helical turn featuring a ribbon representation of β–strands. (**C**) Three connected Ena subunits (as viewed from the central axis of the fiber or exposing the interior) highlighting the jelly roll domain and the *N*-terminal connector (Ntc) (PDB ID 7A02). Arrows point to Cys10, Cys11 of the Ntc from ith subunit that is involved in the disulphide linkage with Cys109, Cys24 of the i-9th and the i-10th subunits, respectively. Reprinted from [29].(**D**) Example of Gram-positive pilin with its Cna domains. Shown here is BcpA, the major pilin of *B. cereus* vegetative pili (PDB ID 3KPT), highlighting the CnaA_2_, CnaA_3_, and Xna domains in light orange, light cyan, and rainbow, respectively [35]. The CnaA domains are composed of two juxtaposed β-sheets of 3–4 strands each, while the Xna domain with a jelly roll topology consists of β-sheets of four to five strands each.

The existence of hair/pilus-like endospore appendages (hereafter called Enas) on spore surfaces was reported already in the 1960s [30,31]. Ankolekar et al. showed that all 47 tested food isolates of *B. cereus* are endowed with Enas. Enas were also found in ten of 12 enterotoxigenic food isolates of *B. thuringiensis* [32]. Whereas the presence of Enas appears to be a general characteristic of *B. cereus* sensu stricto (s.s.) and *B. thuringiensis* spores, they have not been observed on *B. anthracis* or *B. sphaericus* spores [32,33,34], nor on spores of any species belonging to the *B. subtilis* group [34]. Variations in the number of Enas per spore and their morphology have been observed even between strains of the same species. For example, among seven strains of *B. cereus* examined, the number of Enas per spore ranged from one to 23, with an average of five to eight Enas [5], and length ranged from 0.6 to 2 µm [5]. Another strain of *B. cereus* displayed 20–30 Enas per spore, with lengths ranging from 200 nm to 6 µm [29]. Apart from the variation in the length and number of Enas per spore, atomic force microscopy (AFM) of *B. cereus* endospores revealed a thicker type of Enas (ø 8–12 nm, length 0.4–1.2 µm) present together with a thinner type Enas (ø 2.5–3.5 nm, length 0.2–1.6 µm) [28]. Consistent with that, our recent detailed analysis of Enas from the *B. cereus* food poisoning outbreak strain NVH0075-95 using cryo-EM revealed proteinaceous Ena fibers of two distinct morphologies, named staggered (S)- and ladder (L)- Enas [28,29] (Figure 2A). S-Ena is the predominant form of Ena (~90%) in *B. cereus* NVH 0075-95 [29], which presumably corresponds to the thicker type of Ena reported earlier [28]. Interestingly, while the S-Ena appears to be connected to the endospore coat and traverses the exosporium, the L-Ena emerges from the exosporium [28,29] as depicted in Figure 1A.

Although pili have been well-studied structurally and functionally in both Gram-negative and Gram-positive bacteria, spore appendages as a distinct class of pili are just beginning to be characterized. Some of the well-known classes of pili are chaperone-usher pili, type V pili, type IV pili, curli, fap fibers, conjugative, type IV secretion pili, e-pili in Gram-negatives, and sortase-mediated pili, and type IV pili in Gram-positive bacteria [36]. Among Gram-positives, Enas are the third class of pili to be structurally characterized [29]. Until recently, the only endospore appendages whose composition and genetic identity have been characterized were those of *Clostridium taeniosporum* [37]. *C. taeniosporum* has twelve ribbon-like appendages emanating from one pole of the spore. Four proteins, including a glycoprotein, constitute the appendages of *C. taeniosporum* [37]. Notably, no equivalents of these proteins/genes are present among *B. cereus* s.l. spp.

Interestingly, Enas are inherently resistant to boiling (100 °C), autoclavation, desiccation, treatment with strong reducing agents (200 mM β-mercaptoethanol), acids (1 M HCl), chaotropes (8 M urea or 6 M guandinium chloride) and proteases (proteinase K), which hamper mass spectrometry approaches to deduce their amino acid sequences [29,38]. Despite their sturdy nature [29], Ena fibers can be dislodged from the endospore body by sonication [39] or by treatment with sodium thioglycolate [40].

## 4. Architecture of Ena Fibers

The advent of cryo-electron microscopy (cryo-EM) has enabled us to image biological macromolecules down to the atomic level in their native form [41]. It can be used to determine the structure of multiprotein complexes and filaments with known or unknown genetic identities. In our recent work, we used cryo-EM to determine the electron potential map of ex-vivo purified S-Ena appendages at a resolution of 3.2 Å. At this resolution, we could clearly identify amino acids with bulky side chains that led us to recognize a hexapeptide sequence ‘FCMTIRY’ located in the *C*-terminal of an Ena subunit. Searching for this hexapeptide in the *B. cereus* NVH 0075-95 genome revealed the presence of *ena* genes [29]. Ex-vivo purified S-Ena is composed of Ena1A and Ena1B subunits assembled into a helix of 110 Å diameter and 37.4 Å pitch (Figure 2B). Each helical turn is made up of 11.6 Ena1A/B subunits (Figure 2B). Due to their high sequence similarity (identity, 38%; similarity, 58%) and structure, cryo-EM does not help much in unambiguously determining the stoichiometry and arrangement of EnaA/B subunits along the length of the appendages. The Ena1A/B subunits consist of a typical jelly roll fold and a 15-residue long *N*-terminal connecter (Ntc) (Figure 2C). A jelly roll fold generally consists of 8 β-strands arranged in two 4 stranded β-sheets juxtaposed to each other, resembling a jelly or Swiss roll cake [42]. The jelly roll domains of adjacent Ena1A/B subunits are connected to each other through β-sheet augmentation, which helps in the lateral stabilization of the helix (Figure 2B). Apart from that, two complementary electrostatic patches on the surfaces enhance inter-subunit contacts. The Ntc of each subunit is connected to two other subunits present in the preceding helical turn to confer longitudinal stability to the helix. Cys10 and Cys11 in the Ntc of each subunit i form disulphide bonds with Cys109 and Cys24 of subunits i-9 and i-10, respectively. The combination of β-sheet augmentation and covalent linkage through Ntc leads to the assembly of an appendage that is extremely stable, both chemically and physically [29]. Since the Ntcs are present in the luminal side of the helix, they are protected from various environmental assaults. The Ntcs are connected to the jelly roll domain through a flexible five-residue long region that creates a longitudinal gap of 4.5 Å (Figure 2B) between the two helical turns. Due to the flexibility of this spacer region and the lack of direct protein-protein contact between the two subunits present across different helical turns, the appendages turn out to be highly flexible. At the distal termini of the spore, both S-Ena and L-Ena have short extensions that are dubbed ‘ruffles’ (Figure 2A). The S-Ena tends to have four to five ruffles, while the L-Ena has one ruffle per appendage. When observed by negative stain TEM, the ruffles appear morphologically similar to collagen-like immunogenic hairy naps (BclA) that are found attached to the exosporium [43]; however, the protein(s) that constitute the ruffles have not yet been identified.

The jelly roll domain of Ena with its juxtaposed β-sheet architecture has previously been found in many sortase-mediated vegetative pili, such as the XNA domain in BcpA, the major subunit of the vegetative pili of *B. cereus* (Figure 2D) [35]. In addition to that, CnaA and CnaB-type domains found in BcpA (Figure 2D), SpaA pilin from *Corynebacterium diphtheriae* [44], RrgC from *Streptococcus pneumoniae* [45], and FimA and FimP from *Actonomyces oris* [46,47] are another class of domains that contain a sandwiched β-sheet architecture. Cna-type domains are IG-like domains that consist of two juxtaposed sheets. The two sheets can be composed of either 4-4 or 4-5 β-strands (in CnaA) or 4-3 β-strands (in CnaB). These domains were first reported in *Staphylococcus aureus* as collagen-binding proteins [48]. Furthermore, triple jelly roll domains found in the C381 turret protein of *Sulfolobus* turreted icosahedral virus (STIV) are known to be involved in the interaction with the host pilus [49]. All this evidence indicates that the jelly roll domain in Enas might also play a role in adherence to host tissue.

At the domain level, Enas are similar to sortase-mediated pili of vegetative cells of other Gram-positive species. However, the arrangement of these domains in the filament is what distinguishes the two types of pili. Sortase-mediated pili consist of a linear chain of CnaA and/or CnaB domains with distinct genes for basal pilin, backbone pilin, and tip pilin and employ specific sortases to catalyze polymerization and anchoring to the peptidoglycan layer. The sortase-mediated pili rely on internal isopeptide bonds between the side chains of Lys and Asn for thermodynamic and physical stability [50,51]. On the contrary, Ena subunits assemble into a helix and undergo covalent disulphide linkages hidden inside the luminal side to survive extreme physical and chemical stress. Furthermore, Ena1A and Ena1B can self-assemble into filaments nearly identical to S-Ena in vitro [29] without the need for any specific enzyme.

## 5. Ena-Encoding Genes

**Figure 3:**
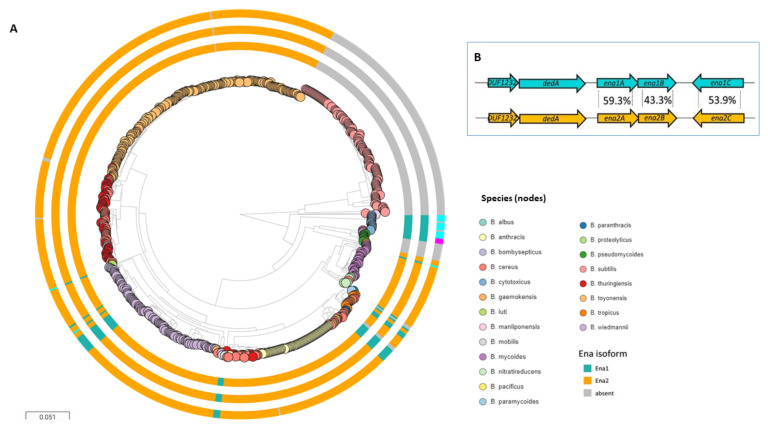
The *ena1* and *ena2* gene loci and distribution of these among 735 *Bacillus* genomes. (**A**) Distribution of *ena1/2A-C* among *B. cereus* s.l. spp. and the presence of genes encoding Ena subunits are indicated on surrounding rings in the following order from inner to outer: presence of *enaA*, *enaB*, and *enaC*, respectively (for all three, *ena1*: teal, *ena2*: orange, different locus: cyan). When no homolog or ortholog was found, the ring is gray. Whole genome clustering of the *B. cereus* s.l. group and *B. subtilis* created by Mashtree [52,53] and visualized in Microreact [54]. Rooted on *B. subtilis*. (**B**) (Inset) Ena1 and Ena2 loci with average amino acid sequence identity indicated between the population of EnaA-C orthologs and homologs found within the *B. cereus* s.l. group. Reprinted from [29].

De novo assignment of an amino acid sequence motif in the electron density map from the atomic model of the S-Ena fibers allowed identification of the protein subunits that build the Enas and the “*ena1*” genes encoding them. The *ena1* genes are located in a gene cluster consisting of *ena1A, ena1B,* and *ena1C*, encoding proteins with theoretical molecular weights of 12, 14 and 17 kDa, dubbed Ena1A, Ena1B, and Ena1C, respectively (Figure 3B). Knocking out any of these three genes produced spores lacking S-Ena, suggesting that all the three protein components are needed for the formation of S-Ena fibers on the surface of the spore [29]. On the other hand, the expression of the L-Ena was not affected by the absence of *ena1* genes, suggesting that the L-Ena fiber is encoded by another currently unknown gene or gene cluster located elsewhere in the bacterial genome [29]. The expression of *ena1A-C* genes was found to be concomitant with endospore formation [29].

## 6. Distribution of Ena within the *B. cereus* s.l. Group

The *ena1A-C* cluster is infrequently found among species belonging to the *B. cereus* s.l. group; only a minority of strains carry homologs of all three Ena1A-C proteins (Figure 3B). Most of these are *B. cereus* and *B. wiedmannii* strains. All *B. cytotoxicus* genomes investigated (*n* = 14/14) carry genes encoding orthologs of Ena1A and Ena1B, and a more divergent Ena1C. The Ena1A and Ena1B proteins in *B. cytotoxicus* and *B. wiedmannii* are slightly different compared to those in *B. cereus* (mean 20% amino acid sequence variation), while the Ena1C is more variable (mean 39.1% amino acid sequence variation, similar synteny). However, a candidate homologous gene cluster is much more common among the *B. cereus* group (~90% of strains) (Figure 3A). This gene cluster encodes proteins, dubbed “Ena2A-C,” that share approximately 40–50% amino acid sequence homology with Ena1A-C and, in most strains, exhibit similar gene synteny as the *ena1A-C* gene cluster in *B. cereus* NVH 0075-95 (Figure 3B). Analysis of more than 700 genomes suggests that nearly all *B. cereus* s.l. strains carry either the *ena1A-C* or the *ena2A-C* gene cluster, but never both simultaneously. The presence of these clusters is, to the best of our knowledge, a unique characteristic of the *B. cereus* s.l. group, as no homologs have been found in other taxa [29]. The finding that the *ena1ABC* or *ena2ABC* gene clusters are widespread among *B. cereus* s.l. species suggests that S-Enas have an important biological function in this group of bacteria [29,31,32]. As members of the *B. cereus* group inhabit a wide range of niches, the observed genetic variability may reflect adaptation to different lifestyles [37].

## 7. Potential Functions of Enas

**Figure 4:**
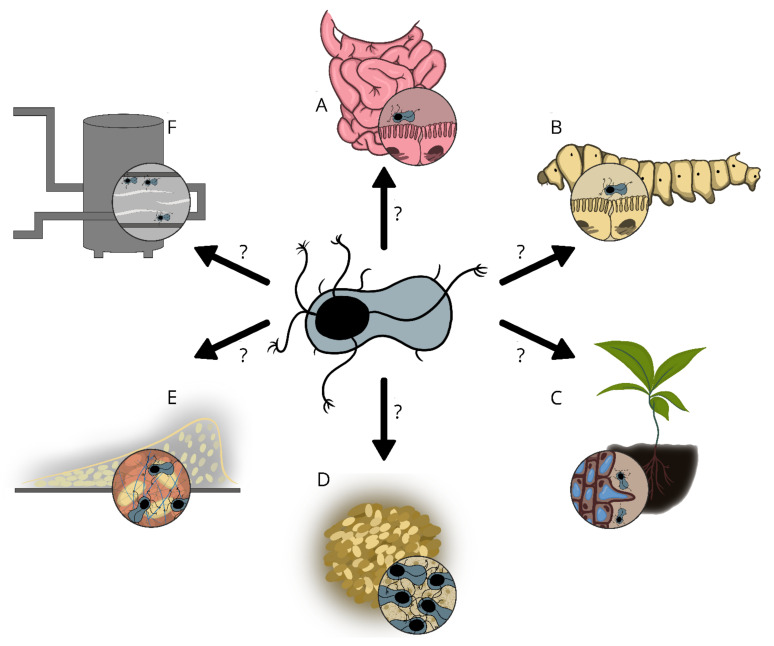
An illustration depicting potential roles that Enas may play in *Bacillus* s.l. spp. Adhesion of endospores to intestinal epithelial cells (**A**), insect tissues (larvae) (**B**), and plant roots (**C**). (**D**) Autoaggregation of endospores. (**E**) Biofilm formation. (**F**) Adhesion of endospores to abiotic surfaces, such as food processing surfaces. The pictures are not drawn to scale.

Although Enas have been known for several decades, their biological function has not yet been unraveled. In cells of Gram-negative, Gram-positive and Archaeal species, pili are involved in a multitude of functions such as adhesion to biotic and abiotic surfaces, exchange of genetic material (conjugation), natural competence, locomotion (twitching motility), biofilm formation, exoprotein secretions, electron transfer (Geobacter) and susceptibility to bacteriophages [55,56,57,58]. Pili are often involved in bacterial adhesion to a diverse range of abiotic and biotic surfaces, such as cells and tissues of plants, animals, or humans. In pathogenic species, pili have repeatedly been shown to contribute to virulence by mediating binding to mucosal surfaces [59]. Although the tip pilins found in sortase-mediated pili, for example SpaC of *Corynebacterium diphtheriae* [60], RrgA of *Streptococcus pneumoniae* [61], FimQ of *Actinomyces oris* [62], etc. are involved in cell-to-cell interaction, it remains to be determined whether Ena ruffles have analogous functions, such as in the interaction of the spores with host receptors. Since pathogenic *Bacillus* s.l. spp. are readily transmitted to their host in the spore form [55], the Enas could play a role in the infection process by, for instance, facilitating the binding of spores to the epithelial cells of the small intestine. Indeed, the presence of ruffles at the distal ends of both S-Ena and L-Ena suggests some roles for Enas in the adhesion and/or recognition of host cell receptors, or abiotic surfaces. Enas are, however, not likely to be involved in active motility or uptake/transport of DNA or proteins, as these are energy demanding processes that are not likely to occur in the endospores’ metabolically dormant state. The potential roles that Enas can play in the various ecological niches of *Bacillus* spp. are summarized in Figure 4.

## 8. Do Enas Function in Adhesion to Abiotic Surfaces?

*B. cereus* spores are capable of adhering to almost all surfaces in food production facilities such as stainless steel, plastic, and rubber, and they are much more adherent compared to their vegetative counterparts [11,63,64]. *B. cereus* biofilms are often found along food and beverage processing lines which causes a great risk of product contamination [9]. The exosporium layer provides *B. cereus* spp. spores with a moderately to highly hydrophobic surface and a net negative charge [32]. This makes them adhere efficiently to hydrophobic surfaces such as stainless steel. It is tempting to speculate that Enas may function in the initial anchoring of the spore to a surface, thereby facilitating short-range interaction between the surface and the spore or the vegetative cell that emerges from the germinating spore [65]. However, data from *different studies have provided contradictory* results on the role of Enas in adhesion to abiotic surfaces, possibly due to the use of different methodologies and strains [3,66,67]. A shear flow-based spore detachment assay comparing four strains of *B. cereus*, *B. thuringiensis*, and *B. pumilus* showed that the presence and number of Enas influences the binding to stainless steel [68]. Interestingly, the study revealed that endospores with a higher number of Enas tend to detach more easily than those with a lower number of Enas [68]. On the contrary, Tauveron and colleagues showed that endospores having many long Enas and a small exosporium tend to adhere better to abiotic surfaces and resist cleaning-in-place (CIP) procedures as compared to spores with a small exosporium and shorter appendages [5]. Endospores of the *B. cereus* group have also been shown to adhere better to hydrophobic surfaces than hydrophilic surfaces, and in some strains, this was attributed to the Enas [64,66]. Furthermore, endospore ultrasonication, which removes appendages from the endospore surface, resulted in reduced attachment of spores to solid surfaces, supporting the hypothesis that Enas play a role in adhesion [66]. In contrast, Stalheim and Granum found that sonication of endospore preparations did not affect the adhesion of spores to stainless steel [39]. Such discrepancies may emanate from the fact that sonication does not only dislodge Enas, but may also partially damage or totally strip off the exosporium from the spore body depending on the intensity of sonication [28]. Since the exosporium is hydrophobic [32,69], a total or partial removal of this layer is expected to alter the adhesion properties of the spores. An alternative explanation could be that the strains they compared may belong to different Ena groups, i.e., Ena1ABC and Ena2ABC, which may exhibit different adhesion properties. To gain a better understanding of the role that S- and L- Enas play in the adhesion of spores to inorganic surfaces, spore adhesion studies involving Ena-depleted mutants are needed. Knowledge from such studies could facilitate the invention of better strategies to prevent the adhesion of spores to stainless steel and other abiotic surfaces in food processing and medical facilities or promote the development of more efficient methodologies for the removal of adhering spores.

## 9. Do Enas Promote the Adhesion of Endospores to Biotic Surfaces?

*B. cereus* s.s. is one of the most common causes of food poisoning incidents, and symptoms include emesis [70] and/or diarrhea [71]. Diarrhea results from the ingestion of spores and vegetative cells followed by the production of various enterotoxins and tissue-destructive hydrolytic enzymes in the small intestine [72,73]. *B. cereus* spores and vegetative cells can adhere to intestinal epithelial cells, which is likely necessary for the onset of diarrheal disease [57,58]. A study of probiotic *B.* *cereus* showed that spores generally adhered better to human enterocytes and mucins compared to their vegetative counterparts [74]. Adhesion to epithelial cells induces spore germination and acts as the first step in colonization, and sometimes development of disease [75]. Although there are reasons to believe that Enas could play a role in the attachment of spores to host tissues, it is currently unknown whether Enas are involved in adhesion to the intestinal epithelium and/or in the virulence of pathogenic *B. cereus* s.l. strains. Among *B. cereus* strains of comparable hydrophobicity, some strains were shown to bind better to Caco-2 cells than others, suggesting that additional factors, presumably Enas, could contribute to cell adhesion [57]. If Enas promote the adhesion of spores to epithelial cells to initiate colonization of host tissue, they could be considered as a virulence factor in pathogenic *B. cereus* s.l. species [57]. It is also tempting to speculate that the variations seen among Enas may, at least in some cases, reflect their host specificity; for example, in the bioinsecticide strains of *B. thuringiensis* [76], Enas may facilitate adhesion of spores to the tissues of insects (Figure 4B). Similarly, as some *B. thuringiensis* strains have plant growth-promoting effects (reviewed in [76]), Enas could play a role in the initial colonization of plant roots (Figure 4C).

## 10. Enas in Autoaggregation and Biofilm Formation

Endospores decorated with Enas were found to stick to each other and form aggregates to a greater extent than spores lacking Enas [28]. Despite being a very common feature among bacteria, the biological function of autoaggreggation is still largely unknown [77]. Some studies suggest that it provides protection against environmental and/or host-associated stresses [77]. It has indeed been shown that autoaggreggation increases spores resistance to heat [78,79], and that non-aggregated spores were more vulnerable to UV irradiation compared to spore aggregates [80]. By playing potential roles in the initial attachment to surfaces and autoaggregation, Enas could be important for initiation of biofilm formation [7]. Previous studies have indicated that amyloid fibers provide backbone and stability to *B. cereus* biofilm matrices [81,82]. As sporulation occurs readily in biofilms [4,9,10], Enas may contribute to the stability of the scaffold matrix formed by amyloid fibers [81,83] as illustrated in Figure 4E. Although structurally different, both Enas and amyloid fibers self-assemble and are extremely resilient fibers [29,81].

## 11. Concluding Remarks

Although the presence of hair-like appendages on the surface of *Bacillus* spp. endospores was reported several decades ago, their biochemical composition, structure, and gene(s) that encode them were unknown until recently. The Enas are suggested to play a role in adhesion to biotic and/or abiotic surfaces, but sufficient experimental data are lacking. As the genes encoding Enas were unknown until recently, efforts to understand their function relied solely on experiments that compared appendage-depleted spores with intact spores. Others have compared strains without considering the existence of different Ena variants, i.e., S-Ena (Ena1 and Ena2) and L-Enas. Both approaches have led to ambiguous data regarding the role of Enas in spore adherence. The recent discovery of the genes encoding the predominant type of Enas (S-Ena) in *B. cereus* s.l. spp. [29], is expected to allow comparative studies involving Ena depleted mutant spores and wild type spores. Such studies are expected to give important insights into the function of these extraordinary fibers in spore adherence to biotic and abiotic surfaces, biofilm formation, spore aggregation, germination, virulence, and other phenomena that would have important implications in the biology of these species. Importantly, knowledge of the potential function of Enas in spore adhesion would also allow the design of more effective strategies to prevent spore binding when harmful or promote binding when beneficial. Additionally, S-type nanofibers, which are highly flexible, and at the same time, inherently resistant to various enzymatic, chemical or heat treatments, may find important applications in nanotechnology.
